# Direct Stimulation of Gastric Smooth Muscle Cells via G_q_ Proteins With Light

**DOI:** 10.1111/nmo.70028

**Published:** 2025-03-31

**Authors:** David Zipf, Markus Vogt, Udhayabhaskar Sathyanarayanan, Ahmed Wagdi, Johannes Riebeling, Robert Patejdl, Tobias Bruegmann

**Affiliations:** ^1^ Institute for Cardiovascular Physiology University Medical Center Göttingen Göttingen Germany; ^2^ Department of Cardiology and Pulmonology, Heart Research Center Göttingen University Medical Center Göttingen Göttingen Germany; ^3^ German Center for Cardiovascular Research (DZHK), Partner Site Lower Saxony, Germany Göttingen Germany; ^4^ Cluster of Excellence “Multiscale Bioimaging: From Molecular Machines to Networks of Excitable Cells” (MBExC) University of Göttingen Göttingen Germany; ^5^ Department of General, Visceral and Paediatric Surgery University Medical Center Göttingen Göttingen Germany; ^6^ Else Kröner Fresenius Center for Optogenetic Therapies University Medical Center Göttingen Göttingen Germany; ^7^ Oscar‐Langendorff‐Institute of Physiology Rostock University Medical Center, University of Rostock Göttingen Germany; ^8^ Department of Medicine Health and Medical University Erfurt Erfurt Germany

**Keywords:** gastric motility, gastroparesis, human Neuropsin (hOPN5), optogenetics, smooth muscle

## Abstract

**Background:**

Optogenetics is a cutting‐edge approach that can enable direct stimulation of gastric smooth muscle cells (SMC) by combining cell‐specific overexpression of light‐sensitive proteins with light stimulation. We previously demonstrated that direct optogenetic stimulation of gastric SMC via depolarization can restore contractility and food propulsion and could become a new treatment strategy for gastroparesis. The human receptor Neuropsin (hOPN5) enables activation of G_q_ signaling with UV light. Herein, we explore this new strategy for direct optogenetic stimulation of gastric SMC.

**Methods:**

We used a transgenic mouse model expressing hOPN5 in fusion with eYFP. Antral longitudinal smooth muscle strips were used for isometric force measurements and whole stomachs for intragastric pressure measurements, comparing light stimulation to other stimuli. Adeno‐associated virus (AAV) serotypes were screened for efficiency in transducing cultured gastric SMC, and transduced cells were tested by Ca^2+^ imaging.

**Results:**

hOPN5 expression was restricted to and found in ~1/3 of SMC in the stomach. UV light induced isometric force and increased intragastric pressure only in transgenic mice similarly to electrical field stimulation and reached approximately 1/3 of the force induced by global depolarization and muscarinic receptor activation. Importantly, optical stimulation remained effective in an ex vivo gastroparesis model. AAV 2.5 was by far the most effective serotype for SMC transduction, and UV light triggered Ca^2+^ transients in SMC expressing hOPN5.

**Conclusion:**

hOPN5 is a new and effective tool to directly stimulate gastric SMC to control contractility with light. Thus, it is an additional and complementary approach to light‐induced membrane depolarization to restore gastric motility.

## Graphical abstract

Optogenetic stimulation enables direct and selective stimulation of smooth muscle cells and thus provides a new opportunity to restore gastric contractility in cases of gastroparesis. Herein, we demonstrate that hOPN5 is a new and effective tool acting via G_q_ signaling and is thus complementary to light‐induced membrane depolarization with channelrhodopsins.

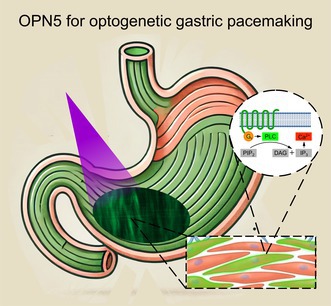

**Summary**

- Optogenetic stimulation enables efficient and direct stimulation of gastric smooth muscle cells.
- Human Neuropsin (OPN5) specifically activates Gq protein signaling and thereby induces Ca^2+^ transients and force generation in smooth muscle cells upon illumination with UV light (~385 nm).
- In the stomach from transgenic mice expressing OPN5 only in the smooth muscle cells, UV light induces intragastric pressure increases independent of the function of the Interstitial Cells of Cajal and the enteric nervous system.
- Thus, direct stimulation of smooth muscle cells with optogenetics can be achieved with a human light‐sensitive receptor providing a complementary approach to channelrhodopsin‐based membrane depolarization.

## 1 Introduction

Direct and selective stimulation of smooth muscle cells (SMC) within the stomach could circumvent the malfunction of the enteric nervous system (ENS) and Interstitial cells of Cajal (ICC) that are leading to dysmotility and gastroparesis [1, 2, 3] and is thus a potential approach to treat this disease. Owing to its complex pathophysiology, treatment of gastroparesis is highly challenging. To this day, none of the multiple existing treatment approaches are effective in directly and specifically targeting the gastric smooth muscle and in precisely controlling gastric contractions to restore motility.

Unfortunately, SMC are far less excitable by electrical field stimulation than neurons and striated muscles, which do express large amounts of voltage‐gated Na^+^ channels. Thus, to electrically stimulate SMC, voltage‐gated Ca^2+^ channels need to be directly excited, which requires depolarization of the membrane potential above −30 mV [4, 5]. In consequence, energy levels required to electrically excite SMC and restore motility would surpass the energy capacity of currently available batteries for stimulation over prolonged periods [6] and can co‐excite surrounding tissue, including the diaphragm and the heart. Consequently, gastric electrical stimulators, which are currently implanted in patients [6] are restricted to low energy levels. They can thus only stimulate ENS, leading to poorly understood afferent signals or efferent responses, which are intended to alleviate symptoms [7]. With active stimulation, gastric emptying is still delayed and the quality of life has not been improved consistently in clinical trials [8, 9, 10, 11]. Therapeutic approaches using prokinetic and antiemetic pharmacological agents act on multiple organ systems and are thus limited by their adverse effects [12]. In conclusion, there remains a significant unmet need for new therapeutic approaches that can preserve gastric function [1, 13, 14, 15].

Optogenetics combines genetic overexpression of light‐sensitive proteins and stimulation with light and thereby allows for the selective stimulation of a certain cell type of interest within an intact organ with high temporal and spatial precision [16]. This new technique has been used to stimulate the ENS of the colon of mice as well as ICC in transgenic mice [17, 18, 19, 20, 21, 22]. We have recently demonstrated that optogenetic stimulation using the nonselective cation channel Channelrhodopsin 2 (ChR2) allows us to directly excite SMC to generate Ca^2+^ transients, induce force, intragastric pressure, and finally restore food propulsion with light [23]. We used transgenic mice expressing ChR2 within the stomach only in SMC and proved that this stimulation is even effective when the ENS and ICC are not intact anymore. Importantly, this direct stimulation of SMC with light was as efficient as global depolarization via high K^+^ levels despite only ~1/3 of SMC expressing ChR2. Since ChR2 has a very low conductance for Ca^2+^ ions at physiological Ca^2+^ concentrations [24], light‐induced Ca^2+^ transients can only be explained by the activation of voltage‐gated Ca^2+^ channels and the generation of action potentials [25]. However, it has been reported that Ca^2+^ channel conductance can be diminished in diabetic gastroparesis [4]. Thus, the efficiency of membrane depolarization mediated optogenetic stimulation could be reduced in diabetic gastroparesis.

Contraction of gastric SMC can not only be induced by action potentials and Ca^2+^ influx from the extracellular site but also via Ca^2+^ released from intracellular stores through IP3 receptors activated by the G_q_ signaling cascade. Neuropsin (OPN5) is a light‐sensitive G protein‐coupled receptor and can be activated by UV light. OPN5 is evolutionarily well conserved from fishes to birds and humans and controls in mammals the local circadian clock in several organs such as the retina, brain, skin, testes, and hypothalamic preoptic area [26, 27, 28, 29, 30, 31, 32, 33, 34, 35]. We and others have proven that it selectively activates the G_q_ signaling cascade [36, 37, 38] and we have been able to demonstrate that human OPN5 (hOPN5) enables UV light‐triggered force generation in the uterus, bladder, and intestine of transgenic mice [36]. Importantly, this G_q_ protein‐mediated force generation is complementary to the membrane depolarization via ChR2, and it has been shown that the response to pharmacological stimulation of G_q_ proteins via acetylcholine binding on muscarinic receptor 3 is not reduced in gastroparesis [39].

Herein, we explore the potency of hOPN5 to control gastric contractility in a transgenic mouse model and screen for potent adeno‐associated virus (AAV) capsids to express optogenetic proteins in gastric SMC.

## 2 Materials and Methods

### 2.1 Transgenic Animal Model

All experiments were in accordance with the European Guideline for animal experiments 2010/63/EU. Transgenic animals expressing hOPN5 in fusion with eYFP under the control of the chicken‐β‐actin promoter, as described previously [36], were crossed into a CD1 background at least seven times. A total of 18 male and 22 female transgenic mice (31 ± 2 weeks) and 10 male and 4 female wild type (WT) animals from the same breeding (22 ± 3 weeks) were used as negative controls. Mice were killed by cervical dislocation, with the exception of the mice used for whole organ experiments, which were killed by decapitation after isoflurane narcosis.

### 2.2 Isolation of Smooth Muscle Cells

Smooth muscle tissue of 4 hOPN5 mice was carefully dissected from the explanted stomachs, cut into small pieces, and placed in ice‐cold Ca^2+^‐free Tyrode solution now supplemented with (in mg/mL): Papain 2 (LS003126, Worthington, USA), Trypsin‐Inhibitor 1 (T9128, Sigma‐Aldrich, Germany), Blend Collagenase 1 (C8051, Sigma‐Aldrich), Collagenase IV‐S 1 (C1889, Sigma‐Aldrich), Fraktion V Albumin 10 (8076.2, Roth, Germany) and Elastase 0.4 (20930.01, Serva, Germany) for at least 30 min at 37°C. The digested tissue was washed, strained through a 200 μm pluriStrainer (pluriSelect, Germany) and stored at 4°C. Cells were imaged within 4 h on glass coverslips. Images were taken using an Olympus IX53 inverted microscope with a Plan N 10×/0.25 Ph1 objective (Olympus, Japan) and an IDS UI‐3040CP‐M‐GL R2 camera (IDS, Germany). Cells were illuminated using an X‐Cite Xylis XT720L LED (Excelitas Technologies, Canada) in combination with a YFP ET Filter Set (Chroma, USA) for an exposure time of 300–1000 ms. To analyze the expression rate, cells were identified and marked in brightfield images, and eYFP fluorescence of the marked areas was normalized to the background signal. Only cells with an eYFP intensity > 8% above the background were counted as hOPN5 positive.

### 2.3 Isometric Force Measurements of Antral Smooth Muscle Strips

Stomachs were immediately explanted and placed in ice‐cold Tyrode solution. Longitudinal smooth muscle strips were cut from the antral muscle layer. Strips were placed in the vertical 20 mL Graz tissue bath (HSE, Germany) filled with Krebs solution (37°C, gassed with 95% O_2_/ 5% CO_2_) supplemented with 5 μM all‐trans retinal (ATR, Sigma‐Aldrich) and fixed between two hooks with a fine thread. Isometric force was measured with FT20 transducers (HSE) connected to a Quad Bridge Amp and a Powerlab 8/35 using the LabChart8 software (ADInstruments, Australia). Strips were pre‐stretched to 2–3 mN and given at least 30 min rest to equilibrate. Global depolarization was achieved with 60 mM KCl (high K^+^) and electrical field stimulation (EFS) was applied in bursts of 50 pulses at 10 Hz with 0.5–1 ms pulse width and 50–100 V by a Universal Isolated Stimulator Output (HSE) via the platinum electrodes integrated into the plastic tissue holders. Illumination was performed with a 385 nm LED within an LEDHub or a HPMOD (Omicron, Germany) controlled by the Omicron software via a 2 mm light guide (NA 0.5) placed orthogonally to the glass wall of the organ bath. Finally, samples were stimulated using 10 μM carbachol (CCh, Sigma‐Aldrich). KCl and CCh were added to the Krebs solution and washed out after a clear peak was reached. After flushing the chambers, the samples were given another 15 min to equilibrate. Responses were measured as the difference between the start of stimulation (if rhythmic spontaneous activity was present the last minimum before start of stimulation) and the maximum force within 1 min after application for KCl and CCh or 20 s after the start of EFS and illumination. To prove G_q_ protein activation by hOPN5, we applied 1 μM YM254890 (Bio‐Techne, USA), a specific blocker for G_q_ proteins [40, 41] and repeated EFS, illumination, and global depolarization after 15 min incubation. To exclude the involvement of direct or indirect neuronal activation, we used 1 μM Tetrodotoxin (TTX, Tocris, UK) and repeated EFS and illumination after 30 min incubation. Responses to stimuli performed multiple times were averaged.

### 2.4 Pressure Measurements of Whole Stomachs

Stomachs were explanted and kept in cooled preparation buffer. A catheter (0.96 mm, Portex, UK) was carefully inserted through the esophagus and pushed forward until the end was placed in the lumen of the antrum. To secure the catheter, a ligature was tied around the base of the esophagus. The catheter was connected to the SP 844 pressure transducer (MEMSCAP, Norway) connected to a PowerLab using the Labchart software8.1.12 (ADInstruments) and perfused with Krebs solution in a 120 mL organ bath filled with Krebs solution (37°C, gassed with 95% O_2_/5% CO_2_) supplemented with 5 μM ATR. Stomachs were given 20 min to equilibrate. Samples were stimulated by global depolarization via 60 mM KCl (high K^+^). EFS was applied in bursts of 59 pulses at 10 Hz with a 1 ms pulse width and 120 V by a Grass‐S8 stimulator (Grass Technologies, USA) via two platinum wire electrodes attached to the sample stage. Global illumination was achieved by three 380–390 nm LEDs (3 W, Avonec, Germany) placed on the sample stage at 120° angles. Furthermore, samples were stimulated using 10 μM CCh.

To mimic gastroparesis, 50 μM methylene blue was applied to the organ bath after CCh stimulation. After 20 min of incubation, the organ bath was flushed once, and the sample was globally illuminated with 630–640 nm LEDs (5 W, Avonec) for 3 min. After an additional 20 min rest, EFS, UV light, and KCl stimuli were repeated. Responses to stimuli performed multiple times were averaged.

### 2.5 Testing of AAV Serotypes

5000 SMC were seeded into each well of a 96‐well plate and incubated for 2 days at 37°C. On the day of transduction, the medium was exchanged for FBS‐free medium (HAMS DMEM/F12 (Sigma‐Aldrich) supplemented with 1% Penicillin/Streptomycin (LifeTechnologies, USA)) and the following AAV were added: AAV 1, 2, 3, 5, 6, 7, 8, 9, 10, eB, and PHP (all containing the CMV‐eGFP construct, from PANEL‐AAVSP01‐20, Vectorbuilder, USA) in increasing concentrations of 1*10^7^, 3*10^7^, 1*10^8^, 3*10^8^, 1*10^9^, 3*10^9^, 1*10^10^, and 3*10^10^ virus particles per well. AAV 2.5 (containing the CAG hOPN5/eYFP gene, produced by SirionBiotech, Germany) was applied in increasing concentrations of 1*10^5^, 3*10^5^, 1*10^6^, 3*10^6^, 1*10^7^, 3*10^7^, 1*10^8^, and 3*10^8^ virus particles per well. Six days afterward, cells were fixed, stained, and imaged as described below. The analysis of expression levels was performed by an individual pipeline in CellProfiler (Broad Institute, USA). Here, a global cut‐off value for pixel intensity was set for both the eGFP/eYFP and the α‐SMA fluorescence signals, which resulted in several representative images with acceptable detection rates of positively stained cells. The cut‐off value was now automatically applied to all images (three per well), and the number of pixels above the specified threshold was determined individually for each channel (GFP or SMA fluorescence) and counted as positive. The expression level in each image was obtained from the ratio of eGFP/eYFP‐positive to SMA‐positive pixels. The results from three associated images were averaged and represent the total expression rate of the respective well.

### 2.6 SMC Transduction and Ca^2+^ Imaging

Cultivated gastric murine SMC were seeded onto 22 × 22 mm glass coverslips coated with Fibronectin 10 μg/mL (F1141‐5MG, Sigma‐Aldrich) in 6‐well plates. For transduction, 50,000 murine gastric SMC were seeded in 1 mL HAMS DMEM/F12 (Sigma‐Aldrich) supplemented with 1% Penicillin/Streptomycin (LifeTechnologies, USA) and 8 × 10^8^ AAV 2.5 (containing the CAG hOPN5/eYFP gene) virus particles (SirionBiotech). After 24 h, 1 mL of HAMS DMEM/F12 supplemented with 10% FBS Superior (Sigma‐Aldrich) and 1% Penicillin/Streptomycin was added, and 1 day later the medium was completely exchanged. For controls, 20,000 SMC were seeded in 2 mL of HAMS DMEM/F12 supplemented with 10% FBS Superior and 1% Penicillin/Streptomycin. On the 4th day after seeding, the cells were used for Ca^2+^ imaging.

Cells were incubated with 2 μM ATR and loaded with 5 μM Calbryte 630 (AAT Bioquest, USA) with 1× PowerLoad Concentrate (LifeTechnologies) in Tyrode solution for 70–80 min at 37°C. Imaging was performed using an inverted IX73 microscope (Olympus) equipped with a 20× objective (LUPCPLFLN20xPH/0.45, Olympus) at 35°C with constant perfusion with Tyrode solution (2 mL/min). Cells were illuminated by an LEDHub (Omicron) equipped with a UV LED (385 nm), a green light LED (500–600 nm) with a 500/20 nm bandpass filter (AHF Analysetechnik AG, Germany) and a red light LED (625 nm) with a 635/18 nm bandpass filter (AHF Analysetechnik AG). The light was transmitted by a 2 mm light guide (NA 0.5) and coupled into the microscope via a dual port microscope coupler (Cairn Research, UK). For imaging of Calbryte 630 fluorescence, red light was directed onto the objective by a BS 409/493/573/652 quadband beamsplitter (AHF Analysetechnik AG) and emission was filtered through a HC 432/515/595/730 quadband filter (AHF Analysetechnik AG). eYFP was excited with green light, and emission collected via a F46‐003 YFP ET‐Filterset (AHF Analysetechnik AG). Images were captured using an Andor Zyla sCOMS camera in combination with the Andor Solis software (Andor, Oxford Instruments, UK). Synchronization of LEDHub and camera timing was done by a PowerLab 8/35 system and the LabChart 8.1.16 Software (ad Instruments).

Cells were stimulated with UV light (385 nm, 100 ms, 240 μW/mm^2^). Only transgenic cells were incubated with 1 μM of YM254890 after the first UV light stimulation, and the ROI was changed. After a 10 min incubation, perfusion was restarted, and the second ROI was also stimulated using UV light. 90 s after the last UV light pulse, both WT and transgenic cells were stimulated with 50 μM Cyclopiazonic acid (CPA, Enzo Life Sciences GmbH, Germany) (flushed in via the perfusion system). For each ROI, an eYFP image was taken after the experiment.

Pictures were analyzed with the ImageJ 1.53c open‐source software (NIH, USA). Maximal F/F_0_ was defined as the highest peak within 20 s after UV light stimulation or 235 s after CPA of every cell. Cells with a baseline intensity of < 10% above background, as well as cells producing < 0.1 F/F_0_ after CPA, were excluded from analysis. Each ROI consisted of at least six cells (hOPN5 *n* = 13, WT *n* = 11) whose max. F/F_0_ was averaged, and the mean of this ROI was taken into account for statistical analysis.

### 2.7 Histology and Immunofluorescence Staining

Explanted stomachs were flushed with cooled Tyrode solution and fixed in 4% formaldehyde for 24 h. The tissue was subsequently placed in PBS (Sigma‐Aldrich) containing 20% sucrose for 48 h. Organs were cryopreserved in TissueTek (Sakura, Germany) and sectioned into 8 μM thick slices using a HM560 cryotome (Thermo Scientific, Germany). Slices were permeabilized using PBS supplemented with 0.2% Triton X (Roth) and 1:1000 DAPI (Th. Geyer, Germany) for 20 min. Primary antibodies were diluted in PBS supplemented with 5% donkey serum (Sigma‐Aldrich) and applied for 2 h at room temperature. Slices were either stained with primary antibodies against β‐III‐tubulin (BioLegend, USA, 802001, 1:800) and c‐kit (Linaris, Germany, MAK5302, 1:400) or α‐smooth muscle actin (A2547, Sigma‐Aldrich, 1:400). Subsequently, secondary antibodies conjugated to Cy3 (711‐165‐152 or 715‐165‐151, 1:400) or Cy5 (712‐175‐153, 1:400) (JacksonLab, USA) were applied together with nanobodies against eYFP (Chromotek, Germany, gb2AF488‐50, 1:400 or NanoTag, Germany, FluoTag‐X4 anti GFP AbberiorStar635P, 1:400) diluted in PBS. Pictures of cryoslices were taken using a Zeiss LSM800 confocal microscope (Zeiss, Germany) equipped with spectral multi‐alkali photomultiplier detectors, a plan apochromat 20× objective, and controlled by the ZEN 3.5 (blue edition) software (Zeiss). The pinhole was set to 1 AU (21 μm for DAPI, 23 μm for Cy2, 30 μm for Cy3, 32 μm for Cy5) and the following channel settings were employed: 405 nm laser and 400–495 nm detection wavelength for DAPI, 488 nm laser and 410–510 nm detection wavelength for Cy2, 561 nm laser and 598–700 nm detection wavelength for Cy3, 640 nm laser and 656–700 nm detection wavelength for Cy5. All channels were used with a 650 V detector gain.

Coverslips used in Ca^2+^ imaging as well as 96‐well plates used for AAV testing were fixed in 4% PFA for 20 min. Coverslips were stained with primary antibodies against α‐smooth muscle actin (SMA) (A2547, Sigma‐Aldrich, 1:400) diluted in PBS supplemented with 0.2% Triton X and 5% donkey serum for 2 h at room temperature. 96‐well plates were first permeabilized with 0.2% Triton X for 20 min before being incubated in PBS supplemented with 5% donkey serum and primary antibodies against GFP (Chromotek 3H9‐100, 1:400) and α‐smooth muscle actin (1:400) for 2 h at room temperature. The secondary antibodies conjugated with Cy5 (712‐175‐153, 1:400, JacksonLab) and diluted in PBS supplemented with 1:1000 DAPI were applied for 1 h at room temperature. Finally, coverslips were mounted on a glass slide using Fluoromount G (LifeTechnologies). Images of coverslips and 96‐well plates were taken using an IX83 inverted microscope (Olympus) equipped with the ORCA‐flash4.0 camera (C11440, Hamamatsu Photonics, Japan) and the MT20 illumination system controlled by the CellSens software (Olympus). Acquisition was performed with a 20× objective (UPLSAPO20X, NA: 0.75) for coverslips and a 10× objective (UPLSAPO10X, NA: 0.4) to analyze AAV transduction efficiency with the following filter settings: 387/11 excitation, 410 beamsplitter, and 440/40 emission for DAPI, 485/20, 504, and 525/30 for eYFP, 560/25, 582, and 607/36 for Cy3, and 650/13, 669, and 684/24 for Cy5.

Pictures of longitudinal antral smooth muscle strips and intact explanted stomachs from transgenic animals were taken using an AxioZoom.V16 microscope (Zeiss) equipped with an Axiocam 705, a plan Z 1.0x objective, and an HXP 200 C light source controlled by the ZEN 3.4 (blue edition) software (Zeiss). A 490–510 nm excitation filter, a 520–550 nm emission filter, as well as a 515 nm beam splitter were used for image acquisition to detect eYFP, and a 559–585 nm excitation filter, a 600–690 nm emission filter, as well as a 590 nm beam splitter for autofluorescence in intact stomachs, and a 450–490 nm excitation filter, a 500–550 nm emission filter, as well as a 495 nm beam splitter for image acquisition to detect eYFP in longitudinal smooth muscle strips. For the intact stomach, individual z‐stacks (with 82–250 μm step size) were stitched together, and the depth of focus was extended via the Zen software. Furthermore, the autofluorescence was subtracted from the eYFP signal.

### 2.8 Solutions

Krebs solution (in mM): NaCl 112, KCl 4.7, CaCl_2_ 2.5, MgCl_2_ 1.2, KH_2_PO_4_ 1.2, Glucose 11.5, NaHCO_3_ 25. Tyrode solution (in mM): NaCl 140, KCl 5.4, CaCl_2_ 1.8, MgCl_2_ 2, Glucose 10, HEPES 10, pH adjusted to 7.4 using NaOH. Ca^2+^‐free Tyrode solution (in mM): NaCl 135, KCl 5, MgCl_2_ 1, Glucose 10, HEPES 10, pH adjusted to 7.4 using NaOH. Preparation Buffer (in mM): NaCl 145, KCl 4.5, CaCl_2_ 0.1, NaH_2_PO_4_ 1.1, MgSO_4_ 1, EDTA 0.025, HEPES 5, pH adjusted to 7.4 using NaOH.

### 2.9 Statistics

Statistical Analysis was performed using the GraphPad Prism 8.2.1 software (GraphPad Software, USA). Aggregated Data is shown as the mean ± standard error of the mean. N represents the number of animals, and n is the number of individual tissue samples or coverslips indicated by the individual dots in the graphs and taken into account for the statistical analysis.

We used a two‐way ANOVA with Sidak's multiple comparison to compare hOPN5 and WT samples in isometric force measurements and intragastric pressure measurements. To compare different stimuli within the hOPN5 or WT group in isometric force measurements and intragastric pressure measurements, a two‐way ANOVA with Tukey's multiple comparison test was used. A two‐way repeated measures ANOVA with Sidak's multiple comparison test was taken to compare the isometric force measurements and intragastric pressure measurements before and after application of either TTX, YM254890 or methylene blue. To determine the EC_50_ values for the AAV expression rate experiments, the GraphPad Prism [Agonist] versus response—Variable slope (four parameters) function provided the best fit and for the light sensitivity analysis the GraphPad Prism [Agonist] versus response (three parameters) function was used. Comparison of light‐induced fluorescence intensity changes of WT SMC, hOPN5 SMC and hOPN5 SMC with YM254890 and comparison of resulting EC_50_ values between AAV were done with an ordinary one‐way ANOVA with Tukey's multiple comparison test. Light‐induced isometric force over a prolonged period was compared using a repeated measures one‐way ANOVA with Tukey's multiple comparison test. Statistical significances are indicated as: *p ≤ 0.05, **p ≤ 0.01, ***p ≤ 0.001, ****p ≤ 0.0001.

## 3 Results

### 3.1 Light‐Induced Force Generation

**FIGURE 1:**
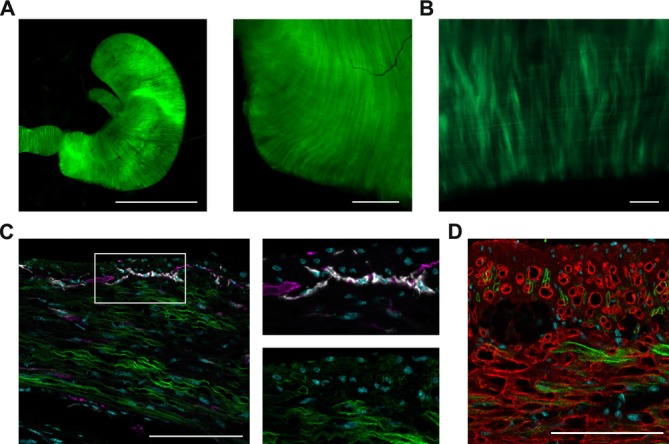
hOPN5 Expression in the stomach of CAG hOPN5/eYFP mice. (A) Overview (left) of an explanted stomach and magnification of the antral part (right) showing the fluorescence signal of hOPN5/eYFP (left: Scale bar = 10 mm, right: Scale bar = 500 μm). (B) Fluorescence image of a longitudinal antral smooth muscle strip showing hOPN5/eYFP (green, scale bar = 200 μm). (C, D) Cryoslices of the gastric antrum colored with blue for nuclei, green for hOPN5/eYFP. c‐kit staining for ICCs is shown in white, β‐III‐tubulin staining for neurons in violet (C). Red is indicating anti‐α‐smooth muscle actin staining for SMC (D). Scale bars = 100 μm.

Transgenic mice expressed hOPN5 in fusion with eYFP under the control of the CAG promoter, which has a strong activity in all muscle cells [36]. Explanted stomachs showed eYFP signal equally distributed over the entire stomach in both the longitudinal and circular muscle layers (Figure 1A,B). On the cellular level, the eYFP signal was restricted to SMC, with no eYFP signal within enteric neurons or ICC (Figure 1C,D) and clearly colocalized with the membranes. Single‐cell isolation revealed eYFP expression in 38% ± 4% (*N* = 4) of SMC isolated from transgenic stomachs.

**FIGURE 2:**
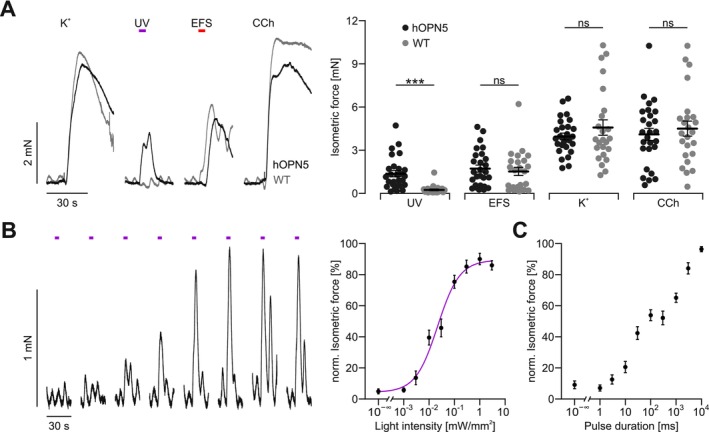
Isometric force measurements of longitudinal antral smooth muscle strips. (A) Representative force traces (left) and aggregated data (right) from hOPN5 (black, *N* = 7, *n* = 27) and WT mice (gray, *N* = 6, *n* = 24). Stimulation was performed with UV light (violet bar, 385 nm, 5 s, 1 mW/mm^2^), EFS (red bar, 10 Hz, 50 pulses, 1 ms, 100 V), global depolarisation by high potassium (K^+^; 60 mM) and CCh (10 μM). Statistical testing with a 2‐way ANOVA test and Sidak's multiple comparison test with the individual results of each strip: *p* (UV light) = 0.0006, all other *p* values comparing WT to hOPN5 mice > 0.75. (B) Representative force traces (left) and aggregated data (right, *N* = 4, *n* = 15) showing the response to UV light illumination (violet bar) with increasing light intensity (left to right: 0.001, 0.003, 0.01, 0.03, 0.1, 0.3, 1, 3 mW/mm^2^) at 5 s pulse length. Aggregated data are shown on the right and light sensitivity was analyzed with a hill fit (violet line). (C) Aggregated data of pulse duration to force relationship using 1 mW/mm^2^ light intensity (*N* = 4, *n* = 15). ***Indicats a *p* value for < 0.0001.

To explore the efficiency of hOPN5 to induce contractions in gastric smooth muscle, we performed isometric force measurements of antral, longitudinal smooth muscle strips and compared illumination with UV light to stimulation with EFS, global depolarization by high K^+^, and muscarine receptor activation by 10 μM CCh. UV light‐induced force was similar to the force after EFS and reached approximately 1/3 of the force induced by global depolarization and muscarine receptor activation. Importantly, strips isolated from WT mice did not react to UV light at all, but the magnitude of force generation was similar for all other stimuli, which excludes functional impairment of SMC due to hOPN5 overexpression (Figure 2A). Interestingly, repetitive illuminations with increasing light intensity or pulse duration showed the ability to precisely control force generation using light (Figure 2B,C) with a half maximal light intensity (eLi50) of 32 ± 7 μW/mm^2^ (*N* = 4, *n* = 15, Figure 2B).

**FIGURE 3:**
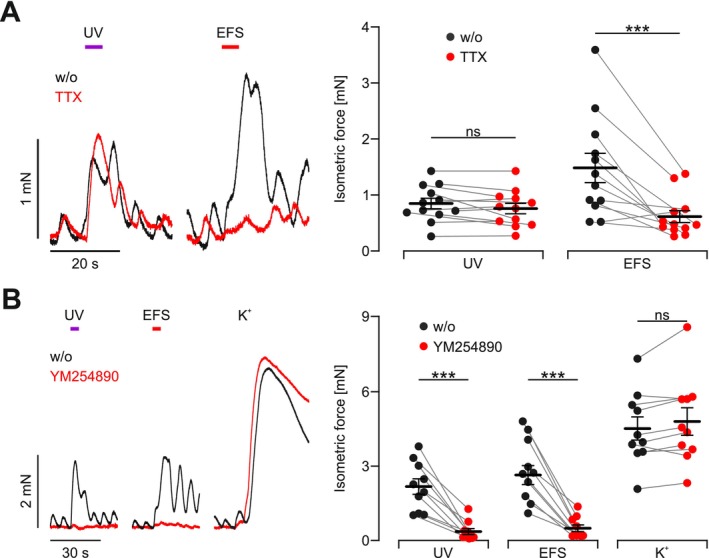
Effects of blocking Na_v_ channels and G_q_ signaling. (A) Representative force traces (left) and aggregated data (right) of isometric force of hOPN5 strips before (black) and after (red) applying 1 μM TTX (*N* = 3, *n* = 12). Contractions were induced by EFS (red bar, 10 Hz, 50 pulses, 1 ms, 50 V) and UV light (violet bar, 385 nm, 5 s, 1.8 mW/mm^2^). Statistical testing with a repeated measures 2‐way ANOVA test and Sidak's multiple comparison test with the individual results of each strip: *p* (UV light) = 0.79, *p* (EFS) = 0.0002. (B) Representative force traces (left) and aggregated data (right) of hOPN5 strips (*N* = 3, *n* = 10) stimulated with UV light (violet bar, 385 nm, 5 s, 1 mW/mm^2^), EFS (red bar, 10 Hz, 50 pulses, 1 ms, 50 V) and global depolarisation by high potassium (K^+^; 60 mM) before (black) and after (red) applying the selective G_q_ protein inhibitor YM254890 (1 μM). Statistical testing with a repeated measures 2‐way ANOVA test and Sidak's multiple comparison test with the individual results of each strip: *p* (UV light) = 0.0006, *p* (EFS) = 0.0002, *p* (K^+^) = 0.28. ***Indicats a *p* value < 0.001.

To exclude co‐activation of the ENS by light stimulation, we applied tetrodotoxin (TTX) blocking voltage‐gated Na^+^ channels [42] which are important for the excitation of neurons but are not expressed in SMC [5]. This nearly completely abolished the effects of EFS, confirming both efficient blockage of the ENS and the neuro‐selectivity of the applied EFS, but had no effect on UV light‐induced contractions (Figure 3A). This proves that UV light directly stimulates SMC with no detectable contribution from the ENS. Applying the highly selective G_q_ protein inhibitor YM254890 [40, 41], light‐induced contractions were reduced by > 80%, whereas responses to global depolarization were unaffected, thus showing that hOPN5‐induced contractions in gastric SMC are mediated by G_q_ signaling (Figure 3B).

**FIGURE 4:**
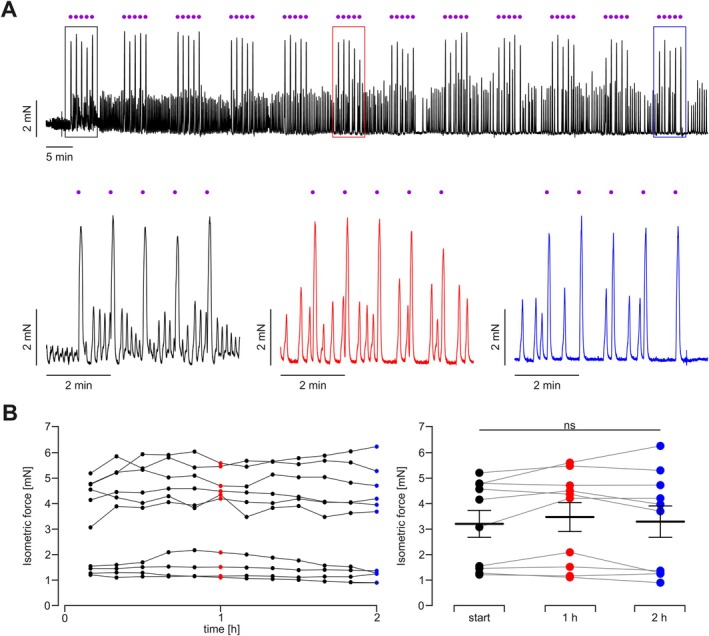
Repeated light stimulation over a prolonged period. (A) Representative isometric force trace of longitudinal, antral smooth muscle strip from a hOPN5 mouse stimulated with UV light (violet dot, 385 nm, 1 s, 0.7 mW/mm^2^) once per minute for 5 min with a 5 min pause after each train of 5. This cycle was repeated 12 times. Shown is an overview of the protocol (top) with a more detailed view of the cycles at the start (black), at 1 h (red) and 2 h (blue) highlighted. (B) Overview (left) and statistical analysis (right) of averaged isometric force of hOPN5 strips over time (*N* = 5, *n* = 10). Statistical testing with a repeated measures one‐way ANOVA test and Tukey's multiple comparison test: all *p* > 0.20.

To test whether repeated light stimulation could lead to desensitization or alteration of function, we applied a prolonged stimulation protocol with repeated light stimulation for 2 h. One illumination cycle comprised five consecutive 1 s long UV light pulses applied once per minute with 0.7 mW/mm^2^ and a 5 min long pause between cycles. We could not detect any change in light‐induced force over this prolonged time period (Figure 4) covering the relevant time period of gastric emptying for the digestible components of mixed meals [43, 44].

### 3.2 Intragastric Pressure Measurements

**FIGURE 5:**
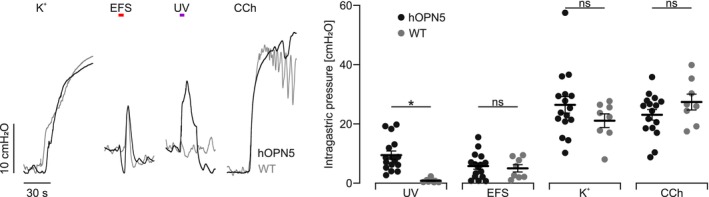
Intragastric pressure measurements. Representative traces (left) and aggregated data (right) of intragastric pressure measurements in intact hOPN5 (*N* = 16) and WT (*N* = 8) stomachs. Stimulation was performed with panoramic UV light illumination (violet bar, 385 nm, 5 s, 0.2 mW/mm^2^), EFS (red bar, 10 Hz, 59 pulses, 1 ms, 120 V), global depolarisation by high potassium (K^+^; 60 mM) and CCh (10 μM). Statistical testing with a 2‐way ANOVA test and Sidak's multiple comparison test: *p* (UV) = 0.01, all other *p* values comparing WT to hOPN5 mice > 0.25. *Indicats a *p* value < 0.05.

To predict how efficiently light‐induced force could restore food propulsion, we performed intragastric pressure measurements of intact, explanted stomachs and compared the effects of UV light stimulation to EFS, high K^+^, and CCh. We performed global illumination with UV light by placing three LEDs panoramically around the explanted stomachs inside the organ bath. Illumination with 0.2 mW/mm^2^ for 5 s induced pressure increases of 9.5 ± 1.4 cm H_2_O, which was ~40% of the pressure induced by global depolarization and muscarine receptor activation. Importantly, the UV light‐induced pressure increase was nearly 4× more compared to EFS. Again, WT stomachs reacted similarly to all stimuli except for UV light illumination, excluding functional impairment due to hOPN5 overexpression also at the whole organ level (Figure 5).

### 3.3 Ex Vivo Gastroparesis Model

**FIGURE 6:**
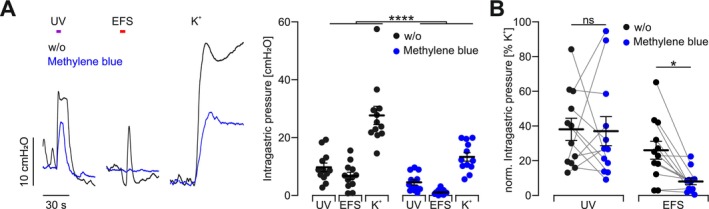
Ex vivo gastroparesis model. (A) Representative traces (left) and aggregated data (right) of intragastric pressure measurements of intact hOPN5 stomachs (*N* = 12) before and after methylene blue phototoxic lesioning. Stimulation performed as described in Figure 4. Statistical testing with a 2‐way ANOVA test: *p* (w/o vs. methylene blue) < 0.0001. (B) Aggregated data of intragastric pressure measurements of intact hOPN5 stomachs (*N* = 12) before and after methylene blue photodynamic lesioning in response to UV light and EFS normalized to the response induced by global depolarisation with high K^+^. Statistical testing with a repeated measures 2‐way ANOVA test and Sidak's multiple comparison test: *p* (UV) = 0.98, *p* (EFS) = 0.023. *Indicats a *p* value < 0.05 and **** for < 0.0001.

Since the ICC and ENS are often dysfunctional but the SMC are still intact in gastroparesis, direct optogenetic stimulation of SMC could provide a new therapeutic option for patients. To demonstrate this principle, we mimicked gastroparesis ex vivo by phototoxic lesioning after methylene blue incubation, which is predominantly taken up by ICC [45, 46]. We observed decreased efficacy for global depolarization by high K^+^, indicating that SMC were also mildly affected. Importantly, EFS acting via the ENS and ICC was not effective at all, whereas UV light was still able to induce pressure increases (Figure 6A). The drop in efficiency was similar for UV light and global depolarization (Figure 6B). This proves that the function of the ICC as an interface between ENS and SMC [47] was completely abolished while the direct stimulation of SMC with hOPN5 and UV light was still effective. Hence, our approach could also be effective in cases of gastroparesis.

### 3.4 Screening for Efficient Gene Transfer

**FIGURE 7:**
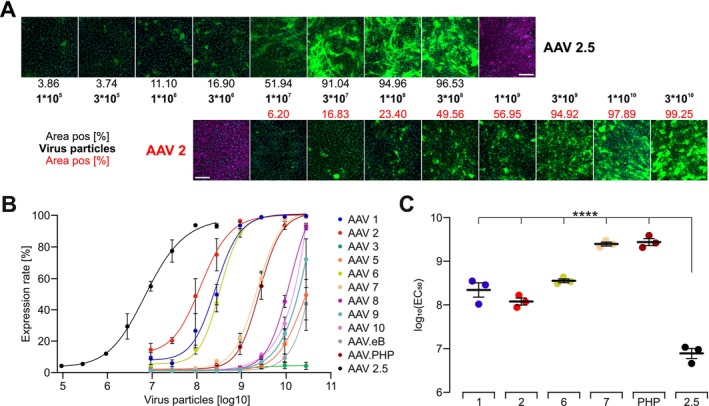
Transduction efficiency of different AAV serotypes in cultivated SMC. (A) Fluorescence images at day 6 post application of AAV2.5 (CAG hOPN5‐eYFP) and AAV2 (CMV eGFP) showing the rate of eYFP positive pixels (green) after applying the indicated number of virus particles. Nuclei are shown in cyan, α smooth muscle actin staining in magenta. Scale bar = 200 μm. (B) Analysis of expression rate in relation to applied virus particles on day 6 after virus application (*n* = 3). (C) Comparison of half‐maximal effective number of applied virus particles [log_10_(EC_50_)] of tested AAV (*n* = 3). Statistical testing with an ordinary one‐way ANOVA with Tukey's multiple comparison test: *p* < 0.0001 for AAV 2.5 versus AAV 1, 2, 6, 7, and PHP. ****Indicats a *p* value < 0.0001.

To overexpress optogenetic receptors in gastric SMC in humans, an efficient gene vehicle is required. Unfortunately, gene transfer into gastric SMC has not been reported so far. AAVs are versatile viral vectors for gene therapy applications, capable of carrying a ssDNA cargo of approximately 4.8 kb, that have been shown to be safe and effective in preclinical and clinical settings, making them a promising option for delivering photoreceptors into target cells. Capsid proteins of individual AAV serotypes use different receptors to enter target cells and thereby create specific tissue/organ tropism for different serotypes [48]. As a first step, we screened for the transduction efficiency of the most common AAV serotypes and AAV 2.5 in cultured SMC isolated from the antrum of WT mice. AAV 2.5 is a synthetic AAV variant created by altering the VP1‐capsid protein of AAV 2 by implementing five mutations from the VP1‐capsid protein of AAV 1 to increase the tropism for skeletal muscle [49] which also led to increased tropism for SMC of blood vessels [50]. All tested AAV serotypes were able to transduce the cultivated SMC, although with varying efficiency. Especially, AAV 2.5 was able to transduce 93% ± 1% (*N* = 3) of SMC at supramaximal concentration and a half maximal effective particle number, which was more than one magnitude lower than any other AAV serotypes (Figure 7).

### 3.5 Ca^2+^ Imaging

**FIGURE 8:**
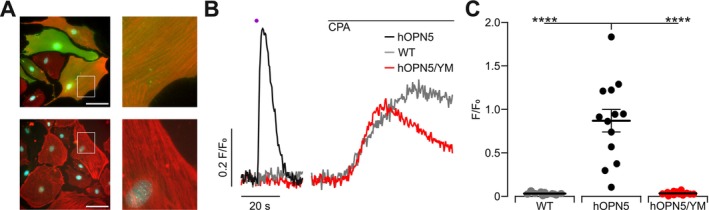
OPN5 Mediated Ca^2+^ transients in WT murine gastric SMC after AAV2.5 transduction. (A) Representative images of hOPN5/eYFP expressing SMC (top) and control SMC (bottom). hOPN5/eYFP signal in green and anti‐α‐smooth muscle actin staining in red, nuclei shown in cyan (bar = 100 μm). (B) Representative Ca^2+^ traces of hOPN5/eYFP SMC, before (black) and after applying 1 μM of YM254890 (red), as well as non‐transduced SMC (WT, gray) after UV light illumination (violet dot, 100 ms, 0.24 mW/mm^2^) and flush in of cyclopiazonic acid (CPA, 50 μM). (C) Aggregated data of maximal light‐induced fluorescence intensity change (F/F_0_) in transduced (*n* = 13) and non‐transduced control SMC (*n* = 11). Statistical testing with an ordinary one‐way ANOVA and Tukey's multiple comparison test: *p* (hOPN5 vs. WT) < 0.0001, *p* (hOPN5 vs. hOPN5/YM) < 0.0001. ****Indicats a *p* value < 0.0001.

To prove that cells expressing hOPN5 after AAV transduction can be stimulated by UV light, we set up all‐optical Ca^2+^ Imaging in SMC visualizing Ca^2+^ alteration with the red‐shifted Ca^2+^ dye Calbryte 630 to avoid excitation of hOPN5 by the imaging light. Upon UV light illumination, SMC expressing hOPN5 showed a clear Ca^2+^ transient, as seen by the fluorescence increase of the Ca^2+^ dye, whereas WT SMC had no reaction to UV light (Figure 8A,B). Applying 1 μM of the highly selective G_q_ protein inhibitor YM254890 completely abolished UV light effects, proving that UV light‐induced Ca^2+^ transients are mediated by G_q_ proteins. Importantly, WT SMC and hOPN5 SMC after YM254890 application showed a Ca^2+^ transient in response to the blocking of SERCA by the application of CPA, showing that they still had intact Ca^2+^ handling (Figure 8B). These experiments prove that AAV selected for SMC tropism can be a highly effective tool for the transduction of gastric SMC and, by combination with hOPN5, enable optogenetic control of previously WT SMC. In additional Ca^2+^ imaging experiments with confocal microscopy, we observed that Ca^2+^ transients in single hOPN5‐positive SMC were not exciting neighboring SMC (Video S1).

## 4 Discussion

Herein, we describe a new method to directly stimulate SMC to control gastric motility by activating G_q_ protein signaling. The human receptor hOPN5 specifically stimulates proteins from the G_q_ protein family [36, 37, 38] which we can prove for the antral strips as well as cultured SMC. We show this mechanism to be highly light sensitive with an eLi50 of ~30 μW/mm^2^, which is approximately three times more light sensitive compared to light stimulation of the ChR2 H134R variant, which could be explained by the intracellular signal amplification of G protein cascades. Light sensitivity is important since UV light (UV‐A, UV‐B, and UV‐C) can have toxic effects due to its DNA‐damaging properties by formation of free radicals and generation of strand breaks [51]. The extent of DNA damage is proportional to the energy content of the applied light probe with UV‐C (< 280 nm) > UV‐B (280–315 nm) > UV‐A (315–400 nm). Nevertheless, UV‐A radiation can also cause smaller amounts of DNA breaks as well as DNA‐protein and interstrand cross‐linking [52, 53, 54]. However, the most effective wavelength to activate hOPN5 (~380 nm) is at the end of the UV‐A spectrum, and hOPN5 can still be activated with blue light (< 450 nm) with reasonable light intensities [36]. Furthermore, the required stimulation frequency and duration for gastric pacemaking (3×/min, for few minutes, 5–7 times a day) and the position of the SMC layer at the outside of the stomach lower the expected required light energy. Within the 2 h long stimulation protocol, we could not detect any difference in contractile response. However, long‐term in vivo experiments are required to determine light energy thresholds for phototoxic effects in the stomach in realistic settings and especially over days, weeks, and months. One advantage of using UV light is low penetration depth and light spreading, potentially minimizing effects on structures and organs in close proximity to the target.

In our experiments, we supplemented the strips and organs with 5 μM all‐trans retinal to achieve reliable light‐induced contractions. The dependence on retinal supplementation has been reported before for hOPN5, likely since mammalian variants can only bind 11‐cis retinal [36, 37, 55, 56] and have a rather high turnover rate requiring fast substitution with new retinal molecules. However, it has been reported that OPN5 variants from nonmammalian species can directly use all‐trans retinal [37, 55, 56]. For example, the expression of the chicken OPN5 allowed in vivo stimulation of cells within the brain without retinal supplementation [37]. In the past, the difference in the kind of retinal binding between pre‐mammalian and mammalian OPN5 variants could be specified to one amino acid exchange during evolution [55]. In the future, this single amino acid exchange could combine the advantage of a predominantly human protein with less dependence on external retinal supply, but this remains to be proven for optogenetic applications. Importantly, the dependence on retinal supplementation, light sensitivity, kinetics, and a redshift of the wavelength for activation could be achieved by single amino acid exchanges, and building chimeras has already been shown for other optogenetic proteins, especially channelrhodopsins. For example, the recently described ChRmine and ChReef already enhanced the efficiency of membrane depolarization‐based stimulation by magnitudes, allowing the heart and brain to be stimulated from outside through the intact body wall [57, 58, 59, 60]. Importantly, using hOPN5 to stimulate G protein signaling is taking advantage of the intracellular signal cascade and its amplification mechanism, which is at least as potent and light sensitive compared to membrane depolarization via channelrhodopsins. Furthermore, the human origin of hOPN5 could become a significant advantage over nonhuman photoreceptors, which are much more likely to trigger an immune response in patients, potentially leading to severe side effects. On the other side, hOPN5 stimulation could only induce 1/3 of the force compared to global depolarization, which corresponds well to the expression rate in the transgenic animal model. This is also in line with the observation that we did not see spreading of Ca^2+^ transients to non‐expressing neighboring SMC. This suggests that G_q_ protein activation stays restricted to the individual cells, which is in clear contrast to depolarization‐based optogenetic stimulation using ChR2 [23].

Furthermore, UV light provides the advantage of better spatial resolution for basic research and could allow better control on sequential stimulation of different parts of the antrum to restore motility in the most physiological pattern. Regarding the demands for gene transfer, depolarization‐based and G protein signaling‐based optogenetic stimulation have opposing demands: While membrane depolarization could overcome low expression rates with high expression strength per cell, hOPN5 would have to be expressed in as many SMC as possible but likely requires less expression per cell due to the signal amplification of the G protein cascade. Our data prove the high efficacy of several AAV capsids, especially AAV 2.5, and that high expression rates can be achieved in vitro. This provides an outlook for further in vivo experiments, including the use of implantable light devices. The latter have been recently demonstrated with successful stimulation for 2 weeks in the bladder [61] and up to 8 days in the colon [62]. Optimizing the optogenetic proteins, efficient gene transfer, and the development of specialized light devices are the key steps toward translation into clinics. Here we demonstrate that using the human receptor hOPN5 is one additional and complementary method with unique advantages.

## Author Contributio ns

D.Z., M.V., T.B., and R.P. designed the experiments. D.Z., M.V., J.R., A.W., U.S., R.P., and T.B. performed and analyzed the experiments. All authors contributed to writing and agreed to the final version of the manuscript.

## Disclosure

Parts of this work have been previously presented at conferences [63].

## Conflicts of Interest

The authors declare no conflicts of interest.

## Supporting information


**Video S1.** Confocal Ca^2+^ imaging with single cell illumination of cultivated murine gastric SMC transduced with an adeno‐associated virus to express hOPN5/eYFP. Representative video of hOPN5/eYFP expressing SMC showing hOPN5/eYFP signal in green and Calbryte 630 Ca^2+^ dye in red. SMC were seeded on Fibronectin coated coverslips and experiments were performed on Day 9 after transduction using a Zeiss LSM800 confocal microscope equipped with spectral multi‐alkali photomultiplier detectors, a plan apochromat 20× objective and controlled by the ZEN 3.5 (blue edition) software. The pinhole was set to 4 AU (104 μm for eYFP, 119 μm for Calbryte 630) and the 405 nm laser was used for hOPN5 stimulation on a small, localized region within the eYFP positive cell indicated by a 10 μm circle (scan speed 10, 38 iterations). Calbryte 630 was imaged with a frame rate of 0.5 Hz after the eYFP was imaged (superposed later on the video).

## Data Availability

The data that support the findings of this study are available on request from the corresponding author. The data are not publicly available due to privacy or ethical restrictions.
